# Development and external validation of a nomogram for predicting the effect of tumor size on survival of patients with perihilar cholangiocarcinoma

**DOI:** 10.1186/s12885-020-07501-0

**Published:** 2020-10-30

**Authors:** Yaodong Zhang, Zhengshan Wu, Xing Wang, Changxian Li, Jiang Chang, Wangjie Jiang, Hongwei Wang, Yirui Wang, Xiangcheng Li

**Affiliations:** 1 Key Laboratory on Living Donor Transplantation, Ministry of Health, Department of liver surgery, The First Affiliated Hospital of Nanjing Medical University, 300# Guangzhou Road, Nanjing, 210029 Jiangsu Province China; 2 Department of Hepatobiliary and Laparoscopic Surgery, Yixing People’s Hospital, Yixing, Jiangsu Province China

**Keywords:** Nomogram, Tumor size, Perihilar cholangiocarcinoma

## Abstract

**Background:**

The effect of tumor size on account of long-term survival results in perihilar cholangiocarcinoma (PCCA) patients has remained a controversial debate. It is urgent necessary to identify the optimal cutoff value of tumor size in PCCA and integrate tumor size with other prognostic factors into a nomogram to improve the predictive accuracy of prognosis of patients with PCCA.

**Methods:**

Three hundred sixty-three PCCA patients underwent surgical resection were extracted from the Surveillance, Epidemiology and End Results (SEER) database. X-tile program was used to identify the optimal cutoff value of tumor size. A nomogram including tumor size was established to predict 1-, 3- and 5-year cancer-specific survival (CSS) based on the independent risk factors chosen by Kaplan-Meier methods and multivariable cox regression models. The precision of the nomogram for predicting survival was validated internally and externally.

**Results:**

PCCA patients underwent surgical resection were classified into 1–19 mm, 20–33 mm and ≥ 34 mm subgroups based on the optimal cutoff for tumor size in terms of CSS. And we noticed that more larger tumor size group had worse tumor grade, advanced T stage, more positive regional lymph nodes and more frequent vascular invasion. The nomogram according to the independent factors was well calibrated and displayed better discrimination power than 7th Tumor-Node-Metastasis (TNM) stage systems.

**Conclusions:**

The results demonstrated that the larger tumor size of PCCA was, the worse survival would be. The proposed nomogram, which outperforms the conventional TNM staging system, showed relatively good performance and could be considered as convenient individualized predictive tool for prognosis of PCCA patients.

## Background

Perihilar cholangiocarcinoma (PCCA) remains one of the most dismal malignancy involving the confluence of the hepatic ducts [1]. Curative-intent resection is still the only effective curative option for PCCA patients that are detected at early stages. However, due to the conceal clinical symptom, most patients are admitted with macrovascular invasion, lymph nodes or liver parenchyma involvement, resulting only 10–15% patients can be amendable to resection with curative intent [2]. Even for the patients underwent surgical resection, the 5-year survival rate remains a disappointing 20–35% [3–8].

Unlike lymph node metastasis and vascular invasion, which have been recognized as the independent prognostic factors in PCCA, the impact of tumor size on account of the long-term survival results in PCCA patients, especially for those who underwent resection, has remained a worldwide controversial debate. Several studies have found no association of tumor size with survival [9–13], while others have reported that tumor size smaller than 3 cm showed better outcome [14–18]. This divergence is also existed in different staging systems. Tumor-Node-Metastasis (TNM) staging system of the American Joint Commission on Cancer (AJCC), which is the wide-accepted standard for PCCA, does not take tumor size into account, whereas the DeOliveira staging system labels tumor size as T1 (1cm), T2 (1-3cm) and T3(≥3 cm) for predicting PCCA patients prognosis [14]. Therefore, we sought to explore the prognostic value of tumor size in PCCA based on data from Surveillance, Epidemiology, and End Results (SEER) Program of the National Cancer Institute.

Due to prognosis prediction is a pivotal reference factor in clinical personalized treatment, a nomogram is an optimal tool, which can incorporate risk factors involved in tumor development, for visually estimating the survival probability of patients based on a statistical predictive model. To our best knowledge, there is no published study reported the nomogram for predicting the prognosis of PCCA based on tumor size. The aims of our study were to evaluate the optimal cutoff value of tumor size in PCCA, and conduct a nomogram incorporated important factors obtained from SEER database for predicting the probability of cancer-specific survival (CSS) of resectable PCCA patients, and externally validating the prognostic model based on the data from the First Affiliated Hospital of Nanjing Medical University.

## Methods

### Data source and selection

**Fig. 1:**
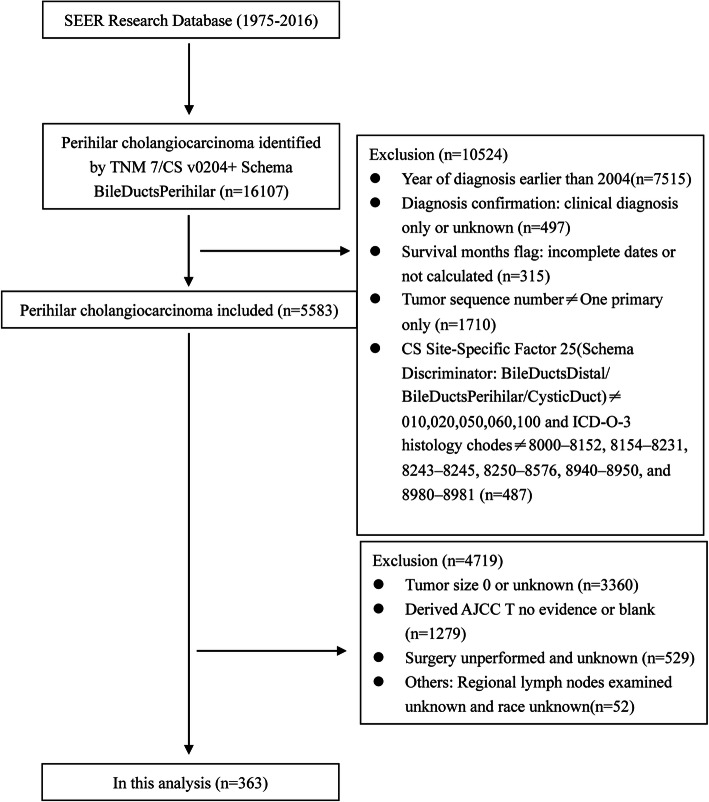
Flowchart displaying the extraction process of resected PCCA cases in SEER database

Data of patients diagnosed with PCCA were obtained from SEER 18 registries research database (1975–2016), which provides cancer incidence and survival information among approximately 30% of the population in the United States. To ensure sufficient time for follow-up and integrity of clinical information, we selected patients included in TNM 7/CS v0204+ Schema BileDuctsPerihilar between 2004 and 2015. On the basis of excluding carcinoid tumor, neuroendocrine carcinoma, and adenosarcoma, we also identified PCCA using ICD-O-3 histology codes reported in Mao et al.: 8000–8152, 8154–8231, 8243–8245, 8250–8576, 8940–8950, and 8980–8981 [19]. And CS Site-Specific Factor 25 (Schema Discriminator: BileDuctsDistal/ BileDuctsPerihilar/CysticDuct) codes 010,020,050,060,100 were applied to further define the location of the tumor in the hilum. Patients were also excluded if they were only diagnosed as PCCA clinically, had incomplete survival time or PCCA was not primary cancer. To explore the prognostic value of tumor size in resectable PCCA, we excluded patients with unknown tumor size, no evidence of primary lesion and not perform the surgical resection. Based on ensuring enough samples for analysis, we excluded patients with incomplete information in such aspects as surgery, regional lymph node detection and race, so as to improve the practical value of nomogram in clinical. The stepwise extraction process of available cases as shown in Fig. 1, a total of 363 cases matching the inclusion criteria were finally chosen in this analysis.

To estimate the generalizability of the novel nomogram based on SEER database. We performed the separate external validation using 132 eligible PCCA cases diagnosed in the First Affiliated Hospital of Nanjing Medical University. The clinicopathological features of all patients are shown in Table 1. The investigation was approved by the ethics committee of the First Affiliated Hospital of Nanjing Medical University.

**Table 1:** Patients characteristics

Characteristic		Training cohort		Validation cohort		*P* value
		No.patients	%	No.patients	%	
Age	< 60	106	29.2	44	33.3	0.376
	≥60	257	70.8	88	66.7	
Race	White	265	73.0			
	Black	26	7.2			
	Others	72	19.8			
Sex	Female	136	37.5	41	31.1	0.204
	Male	227	62.5	91	68.9	
Grade	Well/moderate	217	59.8	61	46.2	< 0.001
	Poor/undifferentiated	102	28.1	68	51.5	
	Unknown	44	12.1	3	2.3	
T stage	T1	55	15.2	8	6.1	< 0.001
	T2	175	48.2	80	60.6	
	T3	50	13.8	30	22.7	
	T4	24	6.6	10	7.6	
	Tx	59	16.3	4	3.0	
M stage	M0	345	95	130	98.5	0.119
	M1	18	5	2	1.5	
RN status	RN examined and not positive	169	46.6	71	53.8	0.253
	RN examined and < 4 nodes positive	114	31.4	40	30.3	
	RN examined and > =4 nodes positive	34	9.4	12	9.1	
	RN not examined or RN unknown	46	12.7	9	6.8	
Vascular Invasion	Absent	315	86.8	84	63.6	0.399
	Present	48	13.2	48	36.4	
Tumor size	1–19 mm	88	24.	29	22.0	0.871
	20-33 mm	160	44.1	60	45.5	
	≥34 mm	115	31.7	43	32.6	

*RN* Regional lymph nodes

### Identification of the optimal cutoff value of tumor size in PCCA

To investigate association of tumor size with survival, X-tile program, which was reported previously [20], was used to divide PCCA patients into three group according to tumor size by log-rank χ2 statistics in terms of CSS. And the optimal cutoff values were validated via internal and external validation.

### Statistical analysis

Information about study cohort collected from SEER database were used to identify the cutoff for the categorical tumor size in terms of survival and develop prognostic nomogram. Nine demographic information and clinicopathological features, including age at diagnosis, race, sex, tumor grade, T stage, M stage (Derived AJCC Stage Group, 7th ed. (2010–2015)), regional lymph nodes status (combined Collaborative Stage Data Collection System (CS) regional nodes examined and CS regional nodes positive), vascular invasion (CS extension) and tumor size (CS tumor size, 2004+) were used to conduct the analysis.

We evaluated the prognostic effect of each clinical variable using cumulative survival curves and log-rank test calculate by Kaplan-Meier method. CSS was set as the main prognostic indicator of patients with PCCA. CSS was defined as the length of time from data of diagnosis to death due to PCCA. Cox proportional hazard models were applied to filter out significant variables, and the independent risk factors chosen by multivariate analysis were incorporated to construct the nomogram for predicting 1-,3- and 5- year CSS. The construct nomogram was subjected internal validation through 1000 bootstrap resamples and further underwent external validated with the 132 patients from the First Affiliated Hospital of Nanjing Medical University. The area under receiver operating characteristic (ROC) curve (AUC) and the consistency index (C-index) were used to verify the precision of nomogram. Meanwhile, calibration plotting was used to evaluate the agreement between the actual outcome and the predicted probability. Decision Curve Analysis (DCA), as a suitable method for evaluating alternative diagnostic and prognostic strategies, was also used to assess the accuracy of nomogram compared to the conventional TNM staging system. All statistical analyses were conducted on SPSS version 21.0 (SPSS Inc., Chicago, IL, USA) and R software 3.6.2 (R foundation, Vienna, Austria). A two tailed *p*-value < 0.05 was considered statistically significant.

## Results

### Patients characteristics and survival outcomes

In total, this study involved 363 eligible patients diagnosed with PCCA as the sole primary cancer and underwent surgical resection in SEER database from 2004 to 2015. The descriptive and clinical characteristics of these patients were provided in Table 1. In the training cohort, the median follow-up periods were 23.87 (range, 1–83) months with 70.8% patients being over age of 60 years. Notably, in this study, we combined the variables (“regional nodes examined” and “regional nodes positive”, which were included in Collaborative Stage Data Collection System) into regional lymph nodes status, which was divided into four subgroups: regional nodes examined and not positive, regional nodes examined and < 4 nodes positive, regional nodes examined and ≥ 4 nodes positive, and regional nodes not examined or regional nodes unknown. 78% resected PCCA patients had less than 4 positive regional lymph nodes. We also noticed that 86.6% patients had no vascular invasion, which was seem as the important factor in prognosis of PCCA. In general, in terms of prognosis, the 1-,3- and 5- year CSS rates were stratified by age, sex, tumor grade, T stage, and M stage (7th edition), regional lymph nodes status, vascular invasion and tumor size.

### Identification of optimal tumor-size cutoff value with prognosis

**Fig. 2:**
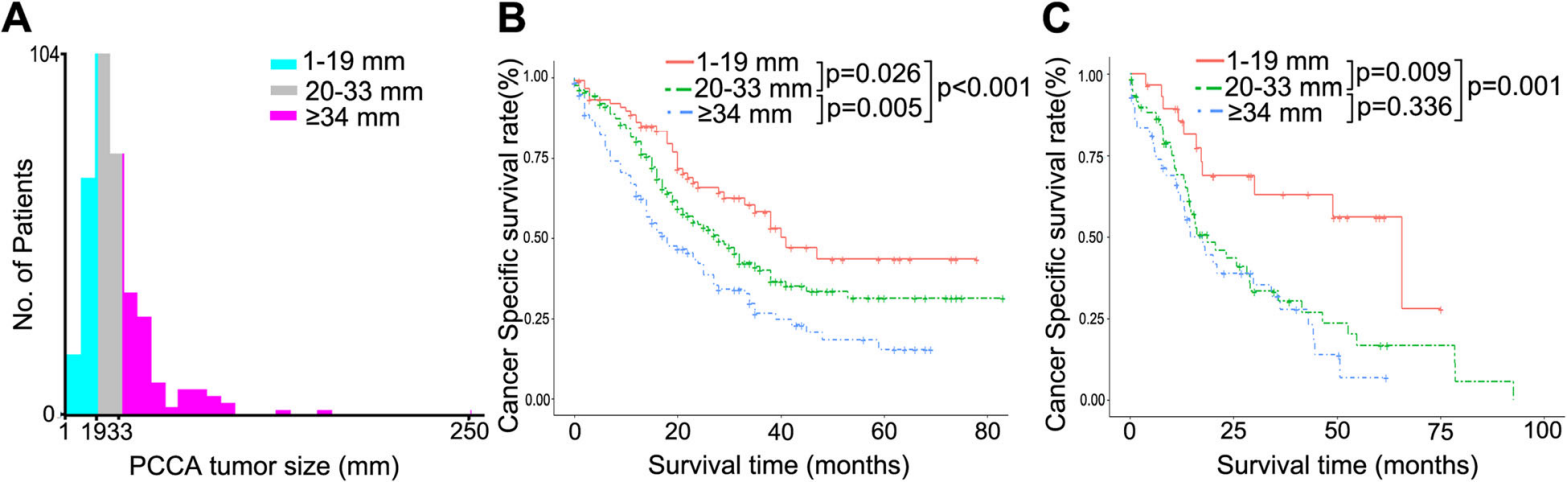
X-tile analysis was done on patient data from the SEER registry. **a** is shown on a histogram of the entire cohort. Cancer-specific survival rates for PCCA patients in training cohort (**b**) and external validation cohort (**c**)

The cutoff values, 20 mm and 34 mm, of tumor size were identified by X-tile plot based on minimal *P*-value approach and the maximum of chi-square log-rank value was 24.1 according to cancer-specific survival (Fig. 2a). To investigate the impact of tumor-size cutoff values on CSS, we first reclassified patients into three risk groups, 1–19 mm, 20–33 mm and ≥ 34 mm, using 20 mm and 34 mm as the cutoff values. The tumor size is an important prognosis factor for PCCA, and patients with 34 mm or more had significant poor prognosis, and 20 mm or less group showed better survival outcome than other groups in CSS (*p* < 0.001, Fig. 2b). In the external validation cohort, we also found that tumor size less than 20 mm in PCCA patients had a better survival outcome, and tumor size larger than 34 mm is a significant negative factor implying a worse prognosis (Fig. 2c). Then, to further confirm the impact of different tumor size on CSS of resected PCCA, we treated the size of PCCA as a continuous variable and analyzed the size of PCCA from 18 to 40 mm (Table 2). The tumor size was the independent factor for PCCA, and patients with tumor size more than 34 mm had significant worse 5-year survival outcome and shorter mean survival time than other groups. Notably, when the tumor size was 33 mm and less, the difference in the prognosis of patients was reduced compared with others. Until the cut-off value was 20 mm, the prognosis of patients with tumor size greater than 20 mm was significantly worse than that with tumor size less than 20 mm. To better reveal the clinical value of these tumor-size cutoff values, we compared the differences in tumor characteristics among the three subgroups. Of these, we noticed that more larger tumor size group had worse tumor grade, advanced T stage, more positive regional lymph nodes and more frequent vascular invasion (Table 3, all *p* < 0.05).

**Table 2:** Univariate analysis for the influence of different cutoffs of tumor size on CSS in perihilar cholangiocarcinoma

Cutoff	No.	5-year CSS (%)	Difference of CSS (%)	Log-rank χ^2^	*P* value	Median of CSS (Months)	Difference of Median (Months)	HR
< 18	71	47.9	23.2	7.407	0.006	41	16	1.705
≥18	292	24.7				25		
< 19	85	42.6	17.7	9.443	0.002	40	16	1.737
≥19	278	24.9				24		
< 20	88	43.5	19.1	11.37	0.001	41	17	1.83
≥20	275	24.4				24		
< 21	122	37.9	13.4	9.716	0.002	38	15	1.617
≥21	241	24.5				23		
< 22	130	37.3	13.1	9.474	0.002	38	15	1.589
≥22	233	24.2				23		
< 23	143	36.4	12.5	10.119	0.001	35	12	1.59
≥23	220	23.9				23		
< 24	152	36.9	13.8	11.094	0.001	35	13	1.614
≥24	211	23.1				22		
< 25	162	36.2	13.1	9.885	0.002	35	12	1.561
≥25	201	23.1				23		
< 26	187	33.9	10	7.067	0.008	33	10	1.477
≥26	176	23.9				23		
< 27	189	35.2	12.8	9.061	0.003	35	12	1.518
≥27	174	22.4				23		
< 28	195	34.9	12.5	9.97	0.002	35	13	1.548
≥28	168	22.4				22		
< 29	200	35.3	13.4	9.969	0.002	33	11	1.547
≥29	163	21.9				22		
< 30	202	34.9	12.7	8.494	0.004	33	11	1.497
≥30	161	22.2				22		
< 31	236	33.6	12.9	8.859	0.003	32	10	1.52
≥31	127	20.7				22		
< 32	240	33.9	14.3	9.037	0.003	32	10	1.529
≥32	123	19.6				22		
< 33	246	35.1	18.5	13.726	0	32	14	1.687
≥33	117	16.6				18		
< 34	248	35.8	20.4	16.741	0	32	14	1.78
≥34	115	15.4				18		
< 35	248	35.8	20.4	16.741	0	32	14	1.78
≥35	115	15.4				18		
< 36	264	34.2	19	13.681	0	32	14	1.715
≥36	99	15.2				18		
< 37	213	35.1	13.6	7.73	0.005	32	9	1.469
≥37	150	21.5				22.5		
< 38	214	34.9	13.2	7.263	0.007	24.8	2.3	1.452
≥38	149	21.7				22.5		
< 39	217	34.4	12.3	5.362	0.021	24.6	1.7	1.379
≥39	146	22.1				22.9		
< 40	277	33	16.4	7.667	0.006	24.9	4.4	1.527
≥40	86	16.6				20.5

**Table 3:** Baseline demographic and tumor characteristics of patients based on tumor size

Characteristic		Tumor size				
		1–19 mm	20-33 mm	≥34 mm	Log-rank	*P* value
Age	< 60	26 (29.5)	48 (30.0)	32 (27.8)	0.16	0.923
	≥60	62 (70.5)	112 (70.0)	83 (72.2)		
Race	White	63 (71.6)	123 (76.9)	79 (68.7)	0.401	4.035
	Black	5 (5.7)	9 (5.6)	12 (10.4)		
	Others	20 (22.7)	28 (17.5)	24 (20.9)		
Sex	Female	29 (33.0)	56 (35.0)	51 (44.3)	3.504	0.173
	Male	59 (67.0)	104 (65.0)	64 (55.7)		
Grade	Well/moderate	53 (60.2)	102 (63.8)	62 (53.9)	10.169	0.038
	Poor/undifferentiated	18 (20.5)	45 (28.)	39 (33.9)		
	Unknown	17 (19.3)	13 (8.1)	14 (12.2)		
T5	T1	24 (27.3)	20 (12.5)	11 (9.6)	28.336	< 0.001
	T2	37 (42.0)	80 (50.0)	58 (50.4)		
	T3	10 (11.4)	21 (13.1)	19 (16.5)		
	T4	2 (2.3)	7 (4.4)	15 (13.0)		
	Tx	15 (17.0)	32 (20.0)	12 (10.4)		
M	M0	85 (96.6)	153 (95.6)	107 (93.0)	1.538	0.463
	M1	3 (3.4)	7 (4.4)	8 (7.0)		
RN status	RN examined and not positive	52 (59.1)	76 (47.5)	41 (35.7)	15.744	0.015
	RN examined and < 4 nodes positive	21 (23.9)	53 (33.1)	40 (34.8)		
	RN examined and > =4 nodes positive	3 (3.4)	15 (9.4)	16 (13.9)		
	RN not examined or RN unknown	12 (13.6)	16 (10.0)	18 (15.7)		
Vascular Invasion	Absent	83 (94.3)	141 (88.1)	91 (79.1)	10.475	0.005
	Present	5 (5.7)	19 (11.9)	24 (20.9)		

### Effect of tumor size counts on survival outcome of PCCA

Initially, univariate analysis revealed that, besides of tumor size, other clinicopathologic characteristics strongly correlated with CSS with tumor grade, T stage and regional lymph nodes status (Table 4). Multivariate analysis with cox regression demonstrated that more larger tumor size showed survival disadvantage in CSS (tumor size: 20-33 mm hazard ratio (HR) 1.525; ≥ 34 mm HR 2.354). Notably, we noticed that tumor size less than 20 mm in PCCA patients indicated a better prognosis, while patients with tumor size larger than 34 mm were a significant negative factor affecting prognosis. Of other variables, tumor grade, T stage and regional lymph nodes status remained significantly associated with CSS in the multivariate cox regression model (all *P* < 0.05), suggesting they were also the independent predictors for CSS in resected PCCA patients (Table 4).

**Table 4:** Univariate and multivariate cancer-specific survival analyses for the influence of tumor size in perihilar cholangiocarcinoma

Variable		Univariable analysis		Multivariable analysis	
		HR (95% CI)	*P*	HR (95% CI)	*P*
Age	< 60	Reference	0.060		
	≥60	1.354 (0.988–1.858)			
Race	White	Reference	0.125		
	Black	1.165 (0.684–1.984)			
	Others	1.419 (1.011–1.991)			
Sex	Female	Reference			
	Male	0.939 (0.708–1.247)	0.666		
Grade	Well/moderate	Reference	< 0.001	Reference	0.003
	Poor/undifferentiated	1.899 (1.401–2.574)		1.733 (1.262–2.379)	
	Unknown	1.255 (0.31–1.897)		1.335 (0.860–2.073)	
T stage	T1	Reference	0.007	Reference	0.170
	T2	1.634 (1.043–2.558)		1.209 (0.756–1.932)	
	T3	1.997 (1.164–3.426)		1.063 (0.599–1.886)	
	T4	2.655 (1.424–4.951)		1.411 (0.735–2.711)	
	Tx	2.324 (1.3753.929)		1.801 (1.032–3.143)	
M stage	M0	Reference	0.063		
	M1	1.676 (0.973–2.887)			
RN status	RN examined and not positive	Reference	< 0.001	Reference	< 0.001
	RN examined and < 4 nodes positive	2.294 (1.658–3.175)		2.168 (1.551–3.031)	
	RN examined and > =4 nodes positive	2.968 (1.857–4.743)		2.592 (1.562–4.302)	
	RN not examined or RN unknown	2.026 (1.317–3.119)		1.952 (1.254–3.039)	
Vascular Invasion	Absent	Reference	0.062		
	Present	1.432 (0.981–2.090)			
Tumor size	1–19 mm	Reference	< 0.001	Reference	0.008
	20-33 mm	1.525 (1.034–2.249)		1.264 (0.844–1.894)	
	≥34 mm	2.354 (1.586–3.494)		1.834 (1.209–2.780)	

*RN* Regional lymph nodes

### Construction and validation of Nomogram for CSS

**Fig. 3:**
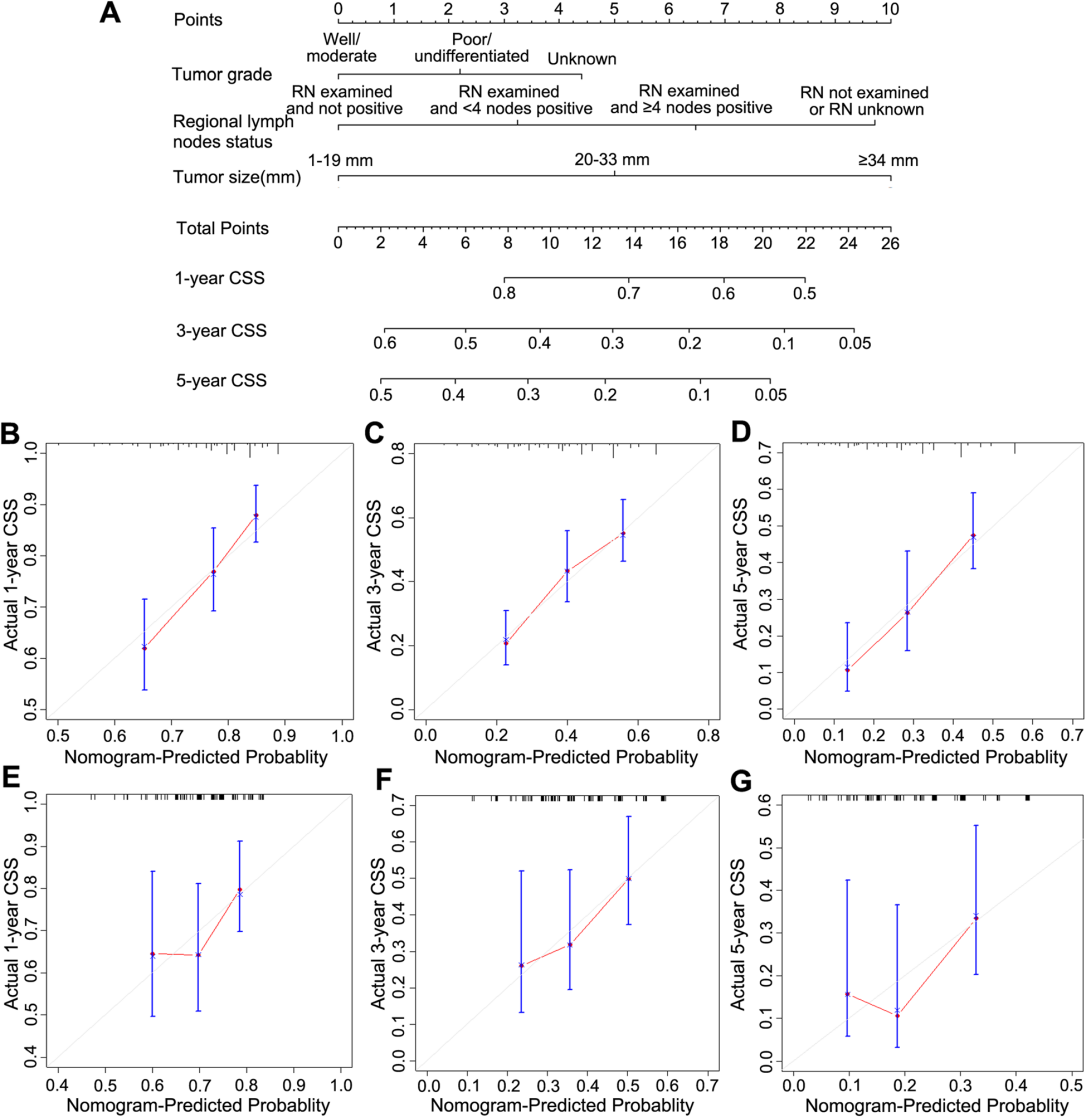
A nomogram predicting 1-,3- and 5-year CSS of patients with PCCA(**a**). Calibration curves for 1-, 3- and 5-year CSS (**b**, **c**, **d**) using nomogram with clinicopathological characteristics and tumor size are shown. External validation using calibration plots for predicting 1-, 3- and 5-year CSS of PCCA (**e**, **f**, **g**)

With the independent predictors of CSS derived from multivariate analysis, we established a nomogram to predict the 1-,3- and 5-year CSS in patients with PCCA. Tumor grade, regional lymph nodes status and tumor size were included into the nomogram (Fig. 3a). Notably, the tumor size contributed the most to prognosis followed by regional lymph nodes status and tumor grade in the novel nomogram. Each variable in the nomogram was assigned a risk score on the point scale. And we were able to calculate the total risk points to estimate 1-, 3- and 5-year survival rates according to the survival probability scale in the nomogram. The nomogram based on tumor size showed good accuracy with c-index of 0.626 for CSS in the training cohort. Calibration plots for the probabilities of 1-, 3-, and 5-year CSS showed the optimal agreement between predictions by the nomogram and the actual observations regarding both training and external validation sets (Fig. 3). To further explore the discriminatory accuracy of the nomogram with that of 7th edition TNM staging systems in the training set. The discriminatory accuracy of the nomogram for CSS prediction was superior to that of 7th edition TNM stage systems (C-index: 0.626 vs 0.608). The external prognostic validation using the above cut-points revealed that prognosis of patients with tumor size less than 20 mm had more better survival outcome, and the calibration plots also performed the good correlations between the nomogram predictions and actual observations for 1-, 3- and 5-year CSS, and C-index of the nomogram was also higher than the 7th edition TNM stage systems (C-index: 0.606 vs 0.541, Fig. 3).

### Comparison of the values of area under the ROC curve

**Fig. 4:**
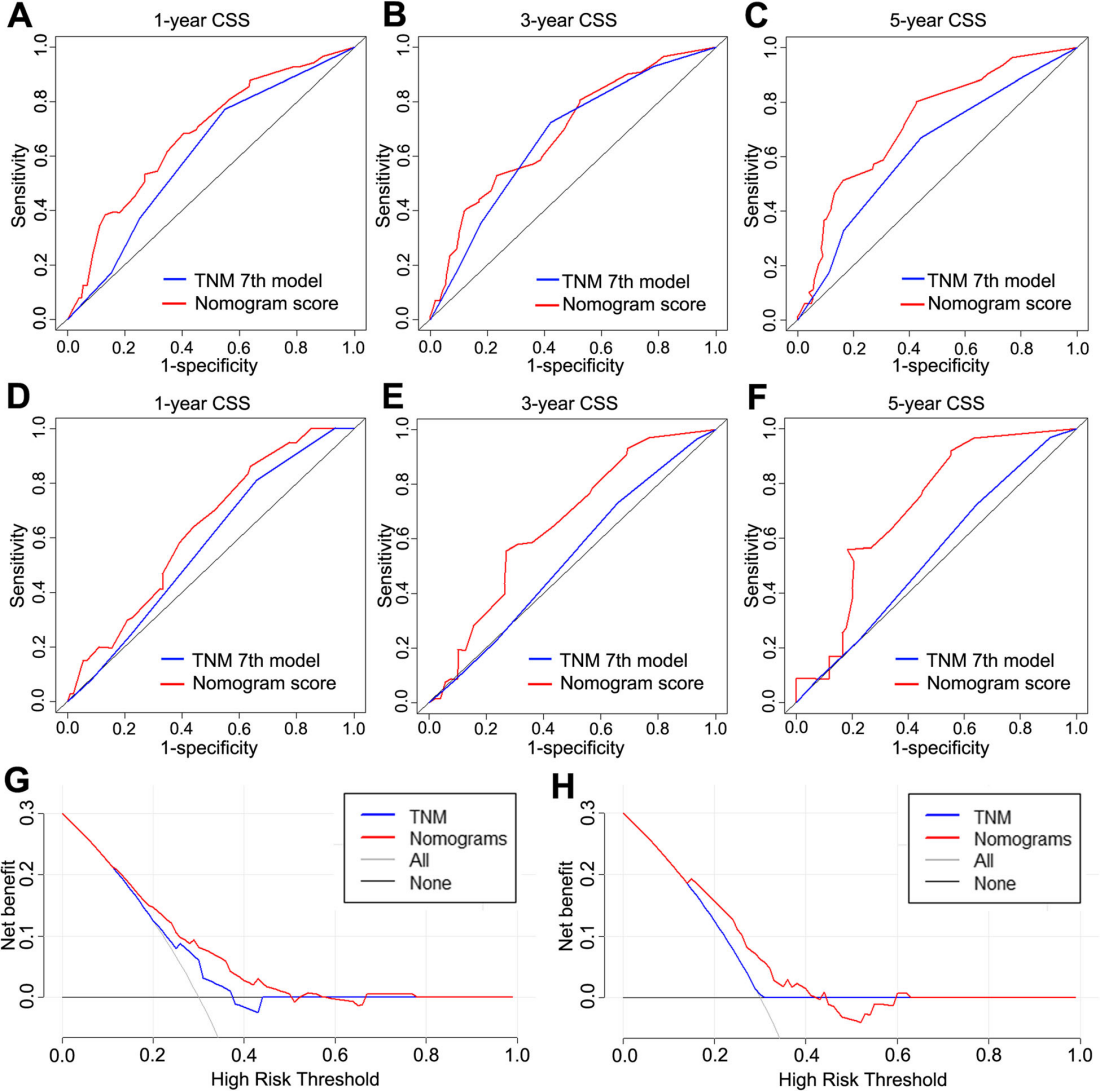
Comparison of the ROC curves of the nomogram and the TNM stage system for 1-,3- and 5-year CSS in training cohort (**a**, **b**, **c**) and external validation cohort (**d**, **e**, **f**). Decision curve analysis of nomogram and TNM stage in patients with PCCA in training cohort (**g**) and external validation cohort (**h**)

Comparison of the novel nomogram and the 7th edition TNM staging system was performed using the AUC values. For the whole study cohort, the AUC values of the nomogram for predicting 1-,3- and 5- year CSS were 0.680, 0.691 and 0.727. These results were significantly better than the corresponding 1- and 5-year AUC values of CSS in TNM staging system (AUC value: 1-year 0.609, 3-year 0.671 and 5-year 0.628, Fig. 4a, b & c). In the external cohort, the1-, 3- and 5-year AUC values of the nomogram also showed better accuracy for predicting the CSS than that with the 7th TNM stage system (0.632 vs 0.572, 0.655 vs 0.527 and 0.709 vs 0.540, Fig. 4d, e & f). DCA was performed to compare the clinical usability and benefits of the nomogram with that of the traditional AJCC stage. As shown in Fig. 4g & h, compared to the AJCC stage model, the new nomogram’s 5-year DCA curves showed larger net benefits across a range of death risk in the training and external validation cohort.

## Discussion

Current curative therapeutic option is limited to surgical treatment which has disappointing outcome with a 5-year survival rate less than 30% [21]. Among the prognostic factors, such as lymph nodal status and resection margin status, influencing the prognosis of PCCA, the impact of tumor size on survival and its clinical value have been still controversial so far. Tumor size was not included in TNM staging system, which is mainly based on the pathological findings. However, different researchers have different views about the effect of tumor size in predicting outcome of PCCA. DeOliveira staging system [22] divided the tumor size into 3 subgroups, T1 (1cm), T2 (1-3cm) and T3(≥3 cm), and emphasized 3-cm cutoff for T3 has worse prognosis compared to smaller tumors [11, 12, 14]. In other studies that supported tumor size should be a prognostic indicator, some researchers suggested 2-cm cutoff is the optimal tumor-size cutoff point.

The nomogram is a visualization tool for calculating the probability of survival for various cancers and has been widely accepted in clinical practice with its feasibility and accuracy [23–25]. Thus, we attempted to establish a prognostic nomogram to predict CSS at 1-, 3- and 5- years in PCCA patients. Via the construction of nomogram, we first used the x-tile program to identify 20 mm and 34 mm as the optimal cutoff value for tumor size in terms of CSS. The results were then further internal and external validated by Kaplan-Meier method to reveal that tumor size within 20 mm showed better prognosis in PCCA patients, and patients with tumors size beyond 34 mm had disappointing survival outcome. Notably, our results demonstrated that more larger tumor size group had worse tumor grade, advanced T stage, more positive regional lymph nodes and more frequent vascular invasion. This finding may implicate that tumor size was closely related to other prognostic factors in PCCA. For the other available prognostic factors by cox regression test involved in the nomogram, tumor grade was demonstrated as an independent prognostic factor, and showed strong impact on CSS of resected PCCA. Meanwhile, given main modification of the number of positive regional lymph nodes in the 8th edition guideline compared to 7th TNM staging system, we set 3 as the cutoff value in positive regional lymph nodes to classify its impact on prognosis. On consistent with the 8th edition guideline, the number of positive regional lymph nodes severed as an independent factor in PCCA. Of note, we for the first time developed the nomogram for assess of the 1-, 3- and 5- year cancer-specific survival based on the tumor size. And our novel nomogram was validated to show the better accuracy than TNM staging system and might to help clinicians to develop personalized treatment strategies to improve the efficiency of medical resource allocation.

It is crucial to determine the generalizability and preventing overfitting of the novel prediction model by internal and external validation. The calibration plots for this study demonstrated the relatively good accurate between the nomogram predicted and actual observed 1-, 3- and 5-year CSS in both the SEER cohort and the external cohort. And compared with TNM stages, our nomogram showed better predictive ability for prognosis of PCCA patients. Remarkably, our nomogram strengthened again the role of tumor size in influencing the survival of PCCA patients underwent resection.

Although this study was based on a large-scale population, there were still several potential limitations. First, SEER database did not record details in other cancer treatments (palliative therapy, radiation, chemotherapy), which play the important roles in the prognosis of resected PCCA. Second, some meaningful manifestations, such as resection margins, liver function and perineural invasion, are not involved in SEER. Third, considering the balance between comprehensibility and comprehensiveness, we only chose the clinical characteristics with high reproducibility and low time-varying.

## Conclusions

In conclusion, we demonstrated that tumor size was an independent factor in predicting the CSS of patients with PCCA. Increased tumor size was associated with worse tumor grade, advanced T stage, more positive regional lymph nodes and more frequent vascular invasion in PCCA. The proposed nomogram, which we constructed and external validated, might be useful to assist clinicians in predicting an individual’s prognosis and planning treatment and follow-up schedules.

## Data Availability

The data that support the findings of this study are available from Surveillance, Epidemiology, and End Results (SEER) Program of the National Cancer Institute (https://seer.cancer.gov).

## Abbreviations

HR: Hazard ratio
PCCA: Perihilar Cholangiocarcinoma
SEER: Surveillance, Epidemiology, and End Results
DCA: Decision Curve Analysis
AUC: The area under receiver operating characteristic curve
RN: Regional lymph nodes
ROC: Receiver operating characteristic
TNM: Tumor-Node-Metastasis
AJCC: The American Joint Commission on Cancer
CSS: Cancer-specific survival
